# Touch to learn: Multisensory input supports word learning and processing

**DOI:** 10.1111/desc.13419

**Published:** 2023-06-08

**Authors:** Amanda H. Seidl, Michelle Indarjit, Arielle Borovsky

**Affiliations:** Speech, Language, and Hearing Sciences, Purdue University, West Lafayette, Indiana,.USA

**Keywords:** ASD, infants, multisensory, perception, touch, word learning

## Abstract

Infants experience language in rich multisensory environments. For example, they may first be exposed to the word applesauce while touching, tasting, smelling, and seeing applesauce. In three experiments using different methods we asked whether the number of distinct senses linked with the semantic features of objects would impact word recognition and learning. Specifically, in Experiment 1 we asked whether words linked with more multisensory experiences were learned earlier than words linked fewer multisensory experiences. In Experiment 2, we asked whether 2-year-olds’ known words linked with more multisensory experiences were better recognized than those linked with fewer. Finally, in Experiment 3, we taught 2-year-olds labels for novel objects that were linked with either just visual or visual *and* tactile experiences and asked whether this impacted their ability to learn the new label-to-object mappings. Results converge to support an account in which richer multisensory experiences better support word learning. We discuss two pathways through which rich multisensory experiences might support word learning.

## 1 | INTRODUCTION

Infants’ early perception and learning is enhanced by sensory information from visual and auditory sources together (e.g., Gogate & Hollich, 2016; Samuelson et al., 2011) that is ever-present in the signals that surround them (e.g., Gogate et al., 2000; Meyer et al., 2011). The richness of this multisensory information goes beyond the visual and auditory to encompass touch (e.g., Nomikou & Rohlfing, 2011; Tincoff et al., 2019; Vigliocco et al., 2019) and even lesser explored word-sense co-occurrences like taste and smell. For example, an infant first being introduced to applesauce by a caregiver may hear the word “applesauce” while simultaneously seeing, smelling, tasting, and touching that applesauce. How might these very rich multisensory experiences impact the ability to learn this word and subsequently to recognize it? It is possible that information from an increasing number of sensory channels may either support or hinder word acquisition and recognition. On one hand, information from an increasing number of sensory channels may serve to “enrich” the encoding of a novel label tied to an object and support its retention and subsequent retrieval. Alternatively, given that multisensory processing capacity increases with maturation (e.g., Lewkowicz, 2014), it is possible that an increasing number of sensory cues may increasingly tax the limited processing capacity of younger children during word-to-world mapping and/or retrieval, thereby slowing or interfering with these processes. For example, the integration of a larger number of multisensory experiences to one percept (e.g., applesauce) may be challenging and recent work suggests that multisensory integration ability is related to associative learning (Barutchu et al., 2020). We explore these questions and alternatives in a series of three experiments. Experiment 1 is an exploratory analysis that asks whether the order of normative vocabulary acquisition is influenced by concepts that have semantic features linked with a greater number of senses. We then follow this initial study with two lab-based empirical experiments which measure whether and how toddlers’ known word recognition (Experiment 2) and novel word learning (Experiment 3) is influenced by the number of sensory channels linked with a word’s meaning.

Our main focus is an exploration of whether and how the number of distinct sensory cues supports or hinders word learning—an idea that has deep roots within both the theoretical history of psychological science and communication sciences and disorders. Specifically, it has long been assumed that access to sensory experience is necessary for acquisition and representation of meaning (Locke, 1690/1948). Further, the relation between sensory experience and representation/learning has been explored through studies of Deaf and blind individuals, who have limited access to one sensory channel. Congenitally blind individuals, who can acquire concepts depicting visually perceptible information (like colors; Kim et al., 2021), may nonetheless also develop representations of concepts that reflect their own sensory experiences with them, such as developing a haptic interpretation of the word “see” (Landau & Gleitman, 1985), or an abstract neural representation for concepts like “rainbow” and “red” (Striem-Amit et al., 2018). Similarly, Deaf children learn language at an age-appropriate time scale when exposed to signed language to which they have full sensory access (in contrast with sensorily inaccessible spoken language; Caselli et al., 2021). Though this work suggests that a range of sensory experiences can alter the ways in which words are learned, this work does not inform us about how the *number* of distinct sensory cues linked with a word impacts learning.

Nonetheless, recent work suggests that links between two sensory cues, that is, audio-visual synchrony, may impact children’s ability to learn words (Gogate, 2022; Gogate et al., 2006; Gogate & Hollich, 2016; Samuelson et al., 2011). For example, in Matatyaho and Gogate (2008) and Gogate et al. (2006), caregivers who display more synchrony between auditory naming and visual object motion have infants who show better word learning. Further, corpus-based studies reveal that the breadth of the child’s sensory experiences enrich the semantic representation of a concept and support word learning. For example, natural languages (in both spoken and signed modalities) exhibit non-arbitrary (iconic) connections between lexical form and meaning (Dingemanse et al., 2015), and that these iconic links have connections with sensorimotor properties of words, with auditory and tactile properties being particularly robust among words that are iconic (Asano et al., 2015; Maurer et al., 2006; Winter et al., 2017). It seems possible that these iconic links may serve to highlight sensorimotor connections between meanings and words, which, in turn, facilitate vocabulary acquisition (Caselli & Pyers, 2017; Imai et al., 2008; Perry et al., 2015, 2021; Sidhu et al., 2022; Thompson et al., 2012). For example, adult learners are better at mapping ideophones (words that include non-arbitrary sound-symbolic relations) from other languages to their intended meaning rather than to their opposite meaning, suggesting that these connections may boost word mapping. Further, mothers of young infants similarly over-represent ideophones in the infant-directed speech, which may support the acquisition of these words and aid in bootstrapping other vocabulary (e.g., Jo & Ko, 2018; for similar discussion see Motamedi et al., 2021). In support of these ideas, words with greater numbers of perceptually-linked semantic features tend to be acquired earlier (Peters & Borovsky, 2019), as do words that are rated more highly on measures of “imageability,” which is rated by how easy it is to bring to mind a mental image of the concept (Hansen, 2017; Ma et al., 2009; Smolik, 2019); and “concreteness” (Braginsky et al., 2019), which is the degree to which a word can be experienced by the senses (Spreen & Schulz, 1966). Together, this work paints a broad picture that sensory experience may support the activation, acquisition, and representation of words in both adults and children.

Some experimental evidence also suggests that such rich multisensory experiences may directly impact word learning. For example, words with greater numbers of semantic features (likely tied to sensory experiences), which describe salient aspects of a word’s meaning, tend to show facilitated semantic processing in a variety of lexical processing tasks in adults (Pexman et al., 2003, 2008; Muraki et al., 2020; Sajin & Connine, 2014; Yap et al., 2012). Similarly, infants benefit from coordinated audio-visual information about speech in language learning tasks such as phoneme identification (Lalonde & Werner, 2019; Teinonen et al., 2008), word segmentation (Hollich et al., 2005), word recognition (Jerger et al., 2009), and word learning (Gogate et al., 2006; Havy & Zesiger, 2017; Nomikou et al., 2017; Zamuner et al., 2021), especially in the first year. However, some multisensory experiments with older children and audio-visual exposures yield equivocal results. In Wellsby and Pexman (2019), 5-year-olds were exposed to differing levels of multisensory experience during word learning using a between-subjects design. Results revealed that multisensory experience did not positively or negatively impact word learning. In contrast, Heisler et al. (2010) and Benham and Goffman (2020) both found that children exposed to greater semantic depth during word learning were better able to learn and articulate novel words since they showed less articulatory variability/more stability when the word was linked with more sensorily rich semantic information (e.g., that it was something that could be squeezed). More recent work goes beyond the audio-visual exposure to examine the impact of a broader range of multisensory signals. For example Schroer and Yu (2022) use head mounted eye-trackers and show that infants’ manipulation of objects with their hands, above and beyond looking at objects during naming, significantly explain variance in word learning. Thus our perspective that touch may impact infant word learning, is motivated by a large body of work on embodied cognition showing that perceptual and motor experience impact word learning within a dynamic systems framework (e.g., Yoshida & Smith, 2008; Yu & Smith, 2012). Further, it is also informed by work with children with speech and language disorders who show sensory differences to typically developing children (e.g., children with ASD; Ben-Sasson et al., 2022) which seem to impact their ability to acquire their lexicon (e.g., Lin et al., 2022; Tenenbaum et al., 2014; Venker et al., 2022). Thus, we explored how number of distinct sensory experiences impact the growth of the lexicon and test the hypothesis that a larger number of multisensory experiences with objects will facilitate children’s learning by enriching their representations.

Caregivers often provide tactile and visual cues during linguistic communication (e.g., auditory, visual, and tactile, Gogate et al., 2000, 2015; auditory and visual, Gogate et al., 2006; Nomikou & Rohlfing, 2011; and tactile-auditory, Abu Zhaya et al., 2017; Tincoff et al., 2019). Such cues are particularly helpful and informative for learning (Gogate et al., 2006; Nomikou et al., 2017). For example, caregivers provide tactile cues which are aligned with word onsets and offsets at a level greater than chance (Abu Zhaya et al., 2017, 2019) as well as tactile cues which are semantically related to words being uttered (e.g., touching the belly while saying the word belly; Tincoff et al., 2019). Further, caregivers present multimodal cues including auditory-visual-tactile signals during novel word naming and also present temporal synchrony between such cues (Gogate et al., 2000). Work in this area suggests that spoken word and visible object-related actions facilitate infants’ mapping of speech signals to objects (e.g., Gogate, 2010; Gogate & Bahrick, 1998; Gogate et al., 2006). These cues could be used by the language learning infant to aid in segmentation of the speech stream (Seidl et al., 2015) and in mapping word forms to word meanings—for example, by increasing attention to cross-modal alignment, deixis, or iconicity (see e.g., Masek et al., 2021 for discussion of how attention creates learning moments).

Given this body of evidence for the potential supportive nature of multisensory cues, including gustatory, tactile, and olfactory cues, in word learning in Experiments 1 and 2 we asked whether the array of multisensory properties of objects could predict lexical acquisition (Experiment 1) and processing (Experiment 2). In Experiment 3, we parametrically manipulated the experience of tactile cues in combination with other sensory channels in word learning.

Across all three experiments, we focused on how the number of distinct sensory experiences linked with a wordform may support acquisition. In the first study, we explored how variance in the normative age-of-acquisition (AoA) of early-acquired nouns may be explained by the number of distinct senses linked with the semantic features of an object. Here, if objects with more linked senses facilitated learning, then we would expect that these words should be learned earlier, on average, compared to words with fewer linked senses. The second study more directly explored whether the number of distinct senses linked with words supports representation of those meanings by measuring whether words with relatively more or fewer senses linked influence the child’s ability to retrieve and recognize labeled objects in an eye-tracked lexical recognition task. Finally, the third study built on the first two by asking whether in a novel word learning task, the number of distinct senses linked with a novel object enhanced learning by systematically varying the number of senses linked.

## 2 | EXPERIMENT 1

As an initial exploration of the general hypothesis that multimodal features supports word learning, we asked how normative AoA of early-acquired nouns relates to the number of unique senses linked with each word. We hypothesized that, if multimodal information supports word learning, the AoA of early-acquired nouns should be negatively associated with the number of senses that are activated by wordforms.

### 2.1 | Methods

#### 2.1.1 | Measuring AoA of early-acquired nouns

**FIGURE 1:**
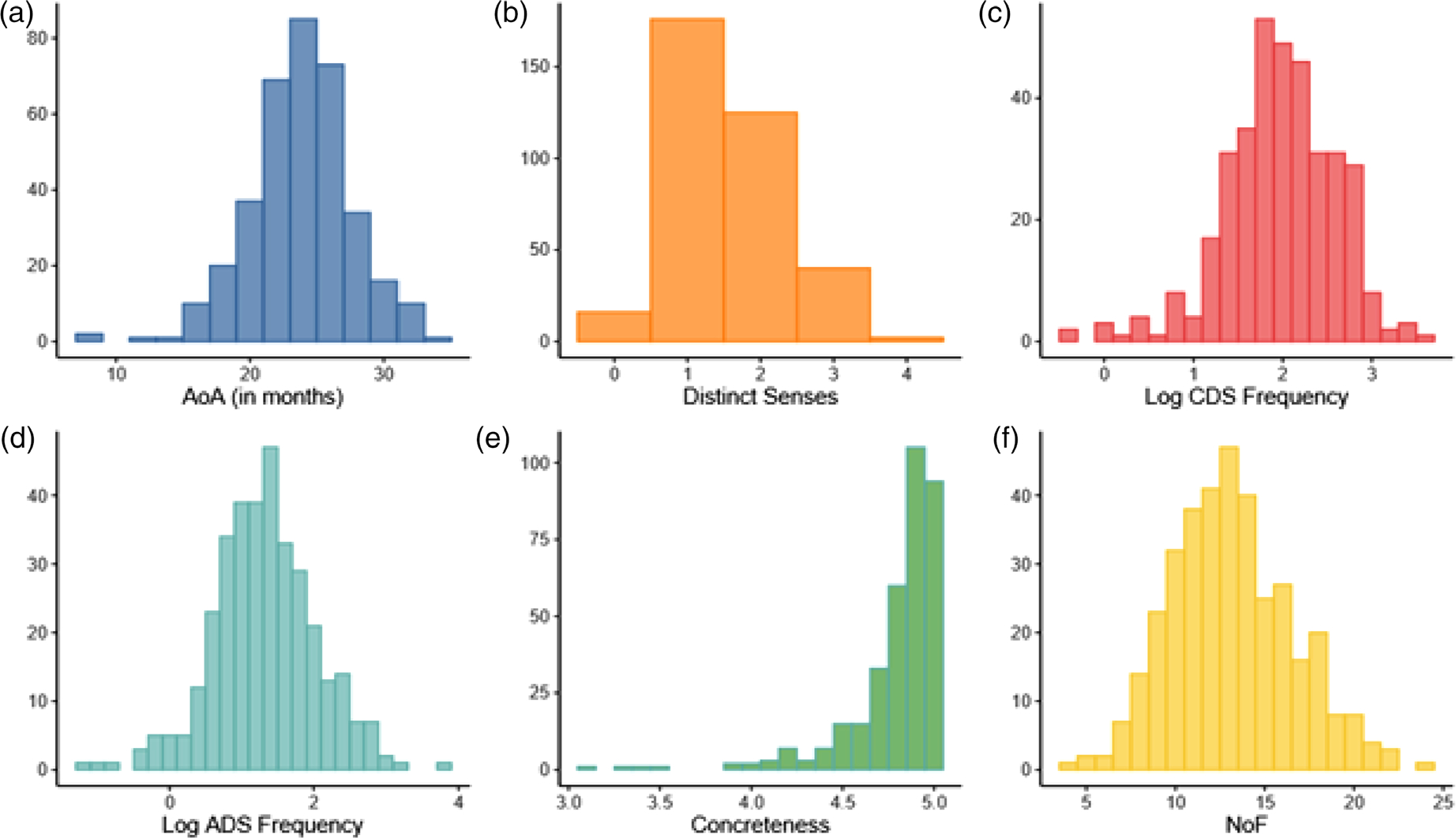
Histograms illustrating distributions of variables across all concepts in the dataset. AoA = Age of Acquisition, Distinct Senses = number of distinct senses associated with each concept, Log CDS Frequency = log of frequency (per million) in child directed speech from the CHILDES database, Log ADS frequency = log of frequency (per million) in adult-directed speech from the SUBTLEX-US corpus, NoF = number of features associated with each concept.

We use the same AoA values calculated from Wordbank data outlined in Peters and Borovsky (2019). In this study, AoA was calculated using vocabulary checklist data from 5450 administrations of the MacArthur-Bates Communicative Developmental Inventory: Words and Sentences (MBCDI:WS) located on Wordbank (Frank et al., 2017). The MBCDI:WS is a popular parental-checklist of early language skills, and is developed for assessing children between the ages of 16–30 months of age. It includes an extensive checklist of words that are produced early by children in this age range. Following Braginsky et al. (2019), Wordbank AoA was calculated using a logistic-curve modeling approach which calculated the proportions of children at each age from 16–30 months who were reported to say each word, and determining the point at which this fitted point crossed 0.5. Two items, “mommy” and “daddy” had a negative intercept (reflecting that these words are typically produced at a very early age and thereby produced by the majority of children by 16 months), and so their AoA was replaced with the first positive month-intercept word, “ball” (8.6).^1^ This AoA estimation procedure yielded values that ranged from 8.6 to 33.5 months (M_AoA_ = 28.8, SD_AoA_ = 3.8). The distribution of AoA is illustrated in Figure 1 (panel A).

#### 2.1.2 | Measuring number of distinct senses linked with early-acquired nouns (distinct senses)

To quantify the number of distinct senses associated with each noun, we used data from an extension of the McRae semantic feature production norms (McRae et al., 2005; part of a larger project currently under development and covers the concrete nouns on the MBCDI:WS). We use the term “semantic feature” to refer to features that include perceptual, sensory, *and* other feature categories (e.g., taxonomic, functional, encyclopedic), and use the term “perceptual features” to describe measures that are derived from perceptual features only.^2^ In these semantic feature datasets, each feature is classified according to a classification system proposed by Cree and McRae (2003). An example of semantic features for a single concept (apple) is illustrated in the second column in Table 1. The subcategory of perceptual features is further sub-categorized (3^rd^ column), as belonging to one of seven *sensory* classes: olfactory, gustatory, auditory, tactile, visual-color, visual-form-and-surface, and visual-motion. In our analyses, we collapsed the three visual feature types (visual-color, visual-form-and-surface, and visual-motion) into a single “visual” sensory category (Perceptual feature column in Table 1). Then, for each of the 359 noun concepts, the number of distinct senses were tallied (for a maximum of 5, see “Number of senses” tally at bottom of Table 1). The distribution ranged from 0 to 4 senses (M = 1.5, SD = 0.8) and is illustrated in Figure 1 (panel B), with most nouns having only one related distinct sensory feature.

#### 2.1.3 | Controlling for variables of frequency (in child- and adult-directed speech), concreteness, number of features

##### Frequency.

Since frequency, concreteness, and number of semantic features have previously been associated with AoA, we sought to gather data from each of these factors for all 359 noun concepts (or as many were available from existing norm sets) in order to control for these variables in our model. Adult-directed speech (ADS) frequency was derived from estimates as the log_10_ values word occurrence per million in the SUBTLEX-US corpus. Frequency estimates were available for 346 nouns (Brysbaert & New, 2009) in this dataset. Child-directed speech (CDS) frequency was estimated as the log_10_ of a word’s frequency (per million words) in the CHILDES database (MacWhinney, 2000), using speech directed towards North American English-learning children at 30 months of age or younger (as existing in the childes-dbversion-0.1.0; Sanchez et al., 2019). The distribution of log_10_ frequency of CDS and ADS across all concepts is illustrated in Figure 1 (panels C and D).

##### Concreteness.

Concreteness ratings reflect the degree to which a word can be experienced through one of the five senses, and the ratings in this study were derived from Brysbaert et al. (2014), which includes ratings for over 40 thousand English words. In this dataset, concreteness ratings were included for the identical form (if it existed) whenever possible, or, if not available, then selected from an alternating plural/singular form. Using this procedure, it was possible to estimate concreteness values for 350 out of 359 nouns on the MBCDI form. Concreteness ratings are measured on a scale of 1 (abstract) to 5 (concrete), and the distribution of concreteness ratings for items in this study are illustrated in Figure 1 (panel E). In general, MBCDI words were rated highly on concreteness, ranging in scale from 3.1 to 5, with a mean of 4.8 and SD of 0.3.

##### Number of features.

The number of semantic features (NoF) was measured as the normative number of semantic features produced for each individual word. This measure is often incorporated into an index of semantic richness of word meaning (Pexman et al., 2008). Here, this measure is included as a control variable to account for the possible confound that words that have a greater number of semantic features (NoF) will also have a greater number of distinct senses linked with them. Across the 359 nouns in this study, NoF ranged from 4 to 24 features (M_NoF_ = 13.1, SD_NoF_ = 3.4). The distribution of NoF is illustrated in Figure 1 (panel F).

### 2.2 | Results

The goal of our first analysis was to measure whether a greater number of distinct senses linked with the components of a word’s meaning predicts the AoA of word meanings. Here, it was hypothesized that words would have an earlier AoA when they are linked with a greater number of distinct senses. We explored this question using a multivariate regression model that models the impact of number of Distinct Senses on AoA, while controlling for frequency (child and adult-directed), concreteness, and number of features. This model is reported in Table 2.

Together, these findings support the hypothesis that earlier acquired words are linked with more distinct senses, even after controlling for potentially confounding variables. The variable estimate of −0.62, indicates that, for every additional sense linked with a concept, the AoA for that concept is reduced by 0.62 months (i.e., ~18.6 days).

In addition to our planned full model, we ran several follow-up analyses to explore alternative explanations for this effect. One possible explanation for this sensory “richness” effect, could be that it is driven by words that have a greater number of *perceptual* features linked with the word’s meaning. To explore this possibility, we ran a second model, and replaced the prior term that included number of features with number of perceptual features only. More specifically, rather than including the total number of features associated with a concept as in the earlier model (which includes features across many sub-types including perceptual, functional, taxonomic and encyclopedic), we only included a total count of features which were classified as perceptual. This number differs from total distinct senses—as number of perceptual features is a summation of all perceptually-related features (and can include multiple visual features, olfactory features, etc…), whereas distinct senses ranges from 0 to 5, depending on whether there is at least a single feature that is categorized as either a visual, auditory, tactile, gustatory or olfactory feature (See Table 1 for a concrete example of how total number of features, number of perceptual features, and distinct senses would apply to an example from a single concept). Again, this model revealed that an increased number of distinct senses contributed to a reduction in AoA of a word, even while controlling for overall number of perceptual features (see Table 3).

In sum, the number of distinct senses associated with a concept is related to a word’s AoA. This result suggests that children may more easily understand and acquire words with meanings linked with more distinct senses. As a first test of this hypothesis, we carried out two experiments to ask whether number of senses relates to better/easier word recognition or word learning: Experiment 2 explored the relationship between number of senses and word recognition and Experiment 3 explored the relationship between number of senses and word learning.

## 3 | EXPERIMENT 2

In Experiment 2 we asked whether the number of senses linked with a known word, impacts the accuracy of its recognition. We predicted that words linked with a larger number of distinct senses would be recognized more quickly and accurately than words with fewer linked senses. Given that we wanted to explore recently learned words in children who were still acquiring much of their vocabulary, but still wanted children to have a range of words that we could test, we chose to enroll children between the ages of 24 and 30 months in this experiment since these children would have a sizable vocabulary of known words which were relatively recently learned, but still be actively engaged in learning many new words.

### 3.1 | Methods

#### 3.1.1 | Participants

Forty-one monolingual English learning children between the ages of 24 to 30 months were invited to take part in the study, which included the experiments described in Experiments 2 and 3 (summary of participant demographics in Table 4). Children were enrolled from a local registry and via flyers placed around a medium sized city in the Midwestern US. Of the 42 who enrolled, eight were excluded for not meeting inclusionary criteria of normal hearing, typical speech/language development, and being monolingual English learners willing to participate in the study. Specifically, four were receiving speech therapy, one was diagnosed with a developmental disorder, and one was hearing a language other than English for more than one hour a day, and one child did not assent to participate in the study (attempting only one trial), while one other’s caregiver reported that their child did not understand any words in the study. Two additional children were removed from the sample as they did not complete at least two trials for every experimental condition. This left a sample of 31 toddlers that contributed data towards the analyses. The study conformed to ethical standards, reviewed and approved by the Purdue University Institutional Review Board, and all caregivers provided informed consent to have their children participate in the study.

### 3.2 | Materials/Experimental stimuli

#### 3.2.1 | Familiar word selection

We used Wordbank (Frank et al., 2017) to select 12 known words—6 of which we classified as high-sensory (hereafter, High words) and 6 of which we classified as low-sensory (hereafter, Low words), with an AoA of, at most, 25-months. Words within the High and Low yoked pairs were also matched closely for AoA (see Appendix A for full list of stimuli, AoA, and yoked pairs used in Experiments 2 and 3) and AoA values across conditions were not significantly different from each other, M_high_(SD) = 19.2 (4.3), M_low_ (SD) = 20.6 (2.7); [t(10) = −0.66, *p* = 0.52]. High words were termed High if they had at *least* three distinct senses linked with them (e.g., the word *banana* triggers auditory, olfactory, gustatory, and visual senses). In contrast, Low words were termed as Low if they had only one non-auditory sensory feature linked (as reported on feature production norm ratings methods described in McRae et al. 2005; e.g., *sky* triggers only visual senses). The 12 words were organized into yoked pairs (e.g., candy-button), with all yoked pairs consisting of a High word (e.g., candy; distinct visual, tactile, and gustatory senses) and a Low word (e.g., button; visual senses only).

#### 3.2.2 | Visual stimuli

Visual stimuli included yoked pairs of photorealistic 400 × 400-pixel color images on a 1920 × 1080-pixel screen. These images were selected to represent prototypical images of the target words. All images were placed on a white background and displayed to the left or right of the screen.

#### 3.2.3 | Auditory stimuli

Auditory stimuli were recorded at a 44.1 kHZ sampling rate by a female native American English speaker in an infant-directed register. Stimuli consisted of the abovementioned known-words (e.g., candy, button), and were followed after a brief delay by tag sentences spoken in an encouraging and child-directed tone (e.g., “great job!,” “Can you find it?”). The use of within-trial tag sentences is frequently reported in the looking-while-listening literature and recommended by a tutorial of this method (Fernald et al., 2008)

Experimental stimuli were adjusted to a mean duration of 800 ms and all stimuli—including the encouraging phrases and an attention-getting word (e.g., Look!) were standardized at a mean intensity of 70 dB in Praat (Boersma & Weenink, 2012).

#### 3.2.4 | Experimental procedure

After the caregiver was consented, we asked each caregiver to complete the MacArthur-Bates Communicative Development Inventories: Words and Sentences (MBCDI:WS; Fenson et al., 2007; Table 4). Then the caregiver and child were brought into the experimental room to start testing with the Looking-while-listening procedure (Fernald et al., 2008) to examine looking behaviors to targets when both High and Low words were played to the infant.

During this procedure participants were seated in a car seat, approximately 60 cm away from the front of a 24-inch monitor and an SR-Research EyeLink 1000 Plus eye-tracking system (SR Research, Ontario, Canada). Caregivers sat slightly behind and to the left of the participant, and an experimenter sat immediately to the right to monitor participants during experimentation, and encourage children to maintain their attention towards the display if they chose to direct their attention to other areas in the room (such as their caregiver). This redirection was only at two time points where trial advancement was contingent on the child’s attention to the screen (described in greater detail below), and only in advance of the critical spoken stimulus. Another experimenter was behind a curtain—out of view from the caregiver and participant—and monitored the eye-tracking equipment and experimental presentation. Caregivers were instructed to refrain from speaking during the procedure.

The eye-tracker was calibrated and focused using a five-point procedure before the experiment began. The five points were represented by a looming bullseye image (30 × 30-pixel) accompanied by a whistling sound on a black background. Post-calibration, a gray screen appeared on the monitor.

**FIGURE 2:**
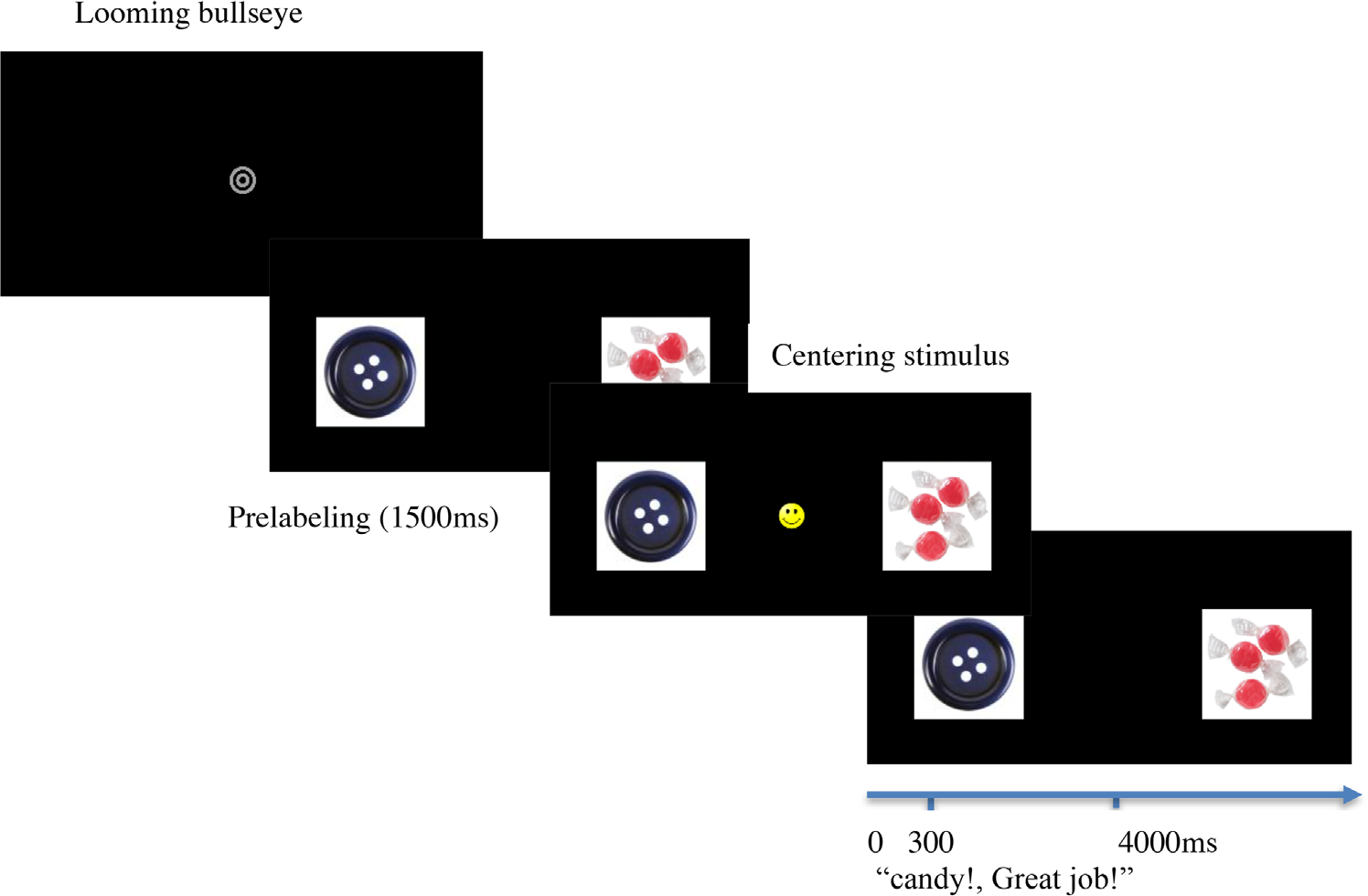
Illustration of one experimental pairing (button & candy) for the procedure of Experiment 2. Gaze dependent labels above photos of procedure and timed actions labeled below.

Another looming bullseye and whistling sound separated each test trial and disappeared once the participants fixated on the bullseye (Figure 2). Immediately replacing the bullseye were the target and distractor images, side-by-side, in a pre-labeling period. After 1500 ms, a salient, centering stimulus appeared on the screen (30 × 30 pixel) between the target and distractor images (e.g., smiley face). Simultaneously, an auditory stimulus, “Look!” was presented. Once the participant looked at the centering image for at least 100 ms, the image disappeared, leaving the target and distractor images. The target’s spoken label was then presented, followed by an encouraging phrase (e.g., “Candy! Great job!”). The target and distractor images were displayed on the screen for 4000 ms, however, the post-labeling test period that we examined lasted from 300 to 4000 ms.

There were 24 test trials containing two types of stimuli (High, Low) with each known High-Low yoked pair (six pairs total) presented on the monitor four times during the experiment and counterbalanced so that each image in the pair appeared as the target image and distractor image twice. Further, all images appeared on each side of the screen the same number of times throughout the experiment. To provide brief breaks and help children maintain their interest and attention across the study, every six trials, children saw images of cartoon characters (such as Winnie the Pooh or Nemo) and heard accompanying (pre-recorded) encouraging phrases like “You’re doing great!.”

Participants’ right eye movements were recorded from image onset to offset at 500 Hz using the SR Research 1000+ eye tracker. These movements were binned into 50 ms intervals for offline analyses. Target and distractor image areas of interest (AOIs) were defined as the 400 × 400 pixel regions comprising the area of each image.

After the experiment, each caregiver was also asked to rate their child’s knowledge of items used in the experiment on a scale from 1 (“child does not say/understand the word”) to 4 (“child says/understands the word”).

#### 3.2.5 | Data cleaning

The final sample of 31 children completed 719 trials across experimental conditions (361 High and 358 Low trials). Following previous research (e.g., Borovsky, 2020), individual trials were removed from further analysis for two reasons: (1) If the child did not yet comprehend the label for the target item as per the caregiver and (2) For excessive track loss. Since the goal of Experiment 2 was to assess toddler’s recognition of familiar words, we asked parents to verify their child’s knowledge of all words in the experiment on a scale from 1 to 4, where “1” indicated that they were very sure that their child did not understand this word, and “4” indicated that they were very sure that their child understands the word. Any target item receiving a rating of less than two was removed from further analysis. This procedure resulted in removing 21 trials from the dataset (2.9% of trials), leaving 698 remaining (351 High, 347 Low). Next, since our goal was to only include trials where children were attentive, and where the eye-tracker was gathering a stable, consistent measure of the eye, we removed trials where more than 80% of total samples over the 4000 ms trial period were either unsampled (defined as either in blink or offscreen). This removal criterion led to the removal of 26 additional trials (3.7%), leaving 672 trials (338 High, 334 Low) that were submitted to the final analysis.

### 3.3 | Results

#### 3.3.1 | Visualizing familiar word recognition

**FIGURE 3:**
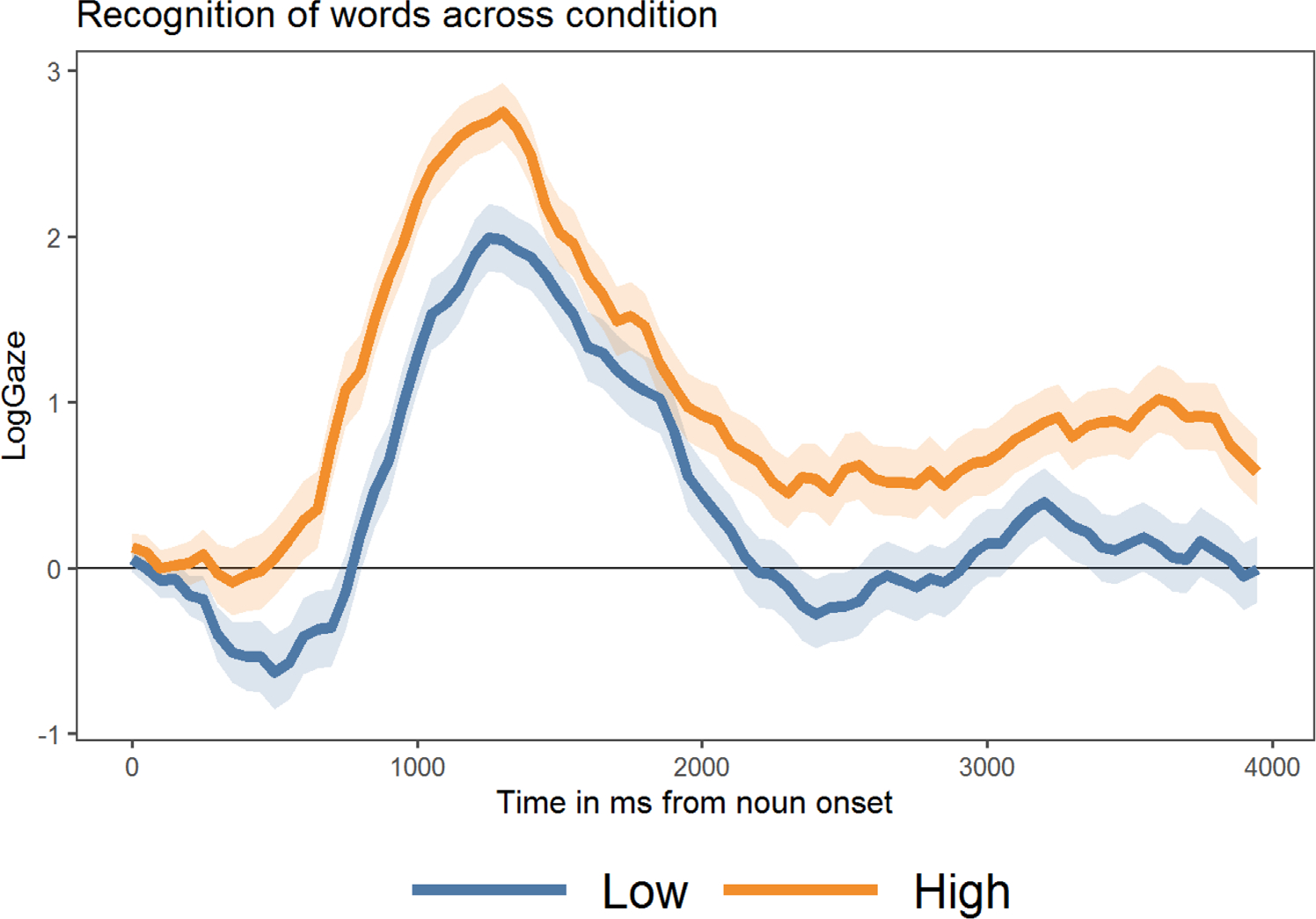
Timecourse of familiar word recognition across high and low sensory words plotted in 50 ms time bins. Positive values indicate a target preference, and negative values indicate a preference to look at the distractor image.

The timecourse of toddler’s recognition as a function of experimental condition is plotted in Figure 3. The rise in positive LogGaze fixation proportions within the first 500–1000 ms after the spoken word onset indicates that, as expected, toddlers rapidly recognized the spoken labels and directed their gaze towards the appropriate target image. This plot also illustrates a clear difference in the timing for High and Low target items—such that it appeared that items in the Low sensory condition took longer for toddlers to uniquely identify from the object array (as indicated by the differences in timing for when High and Low sensory condition show positive LogGaze values). Additionally, these plots illustrate a difference in target recognition between conditions that persisted through much of the plotted time period.

#### 3.3.2 | Time window analysis

**FIGURE 4:**
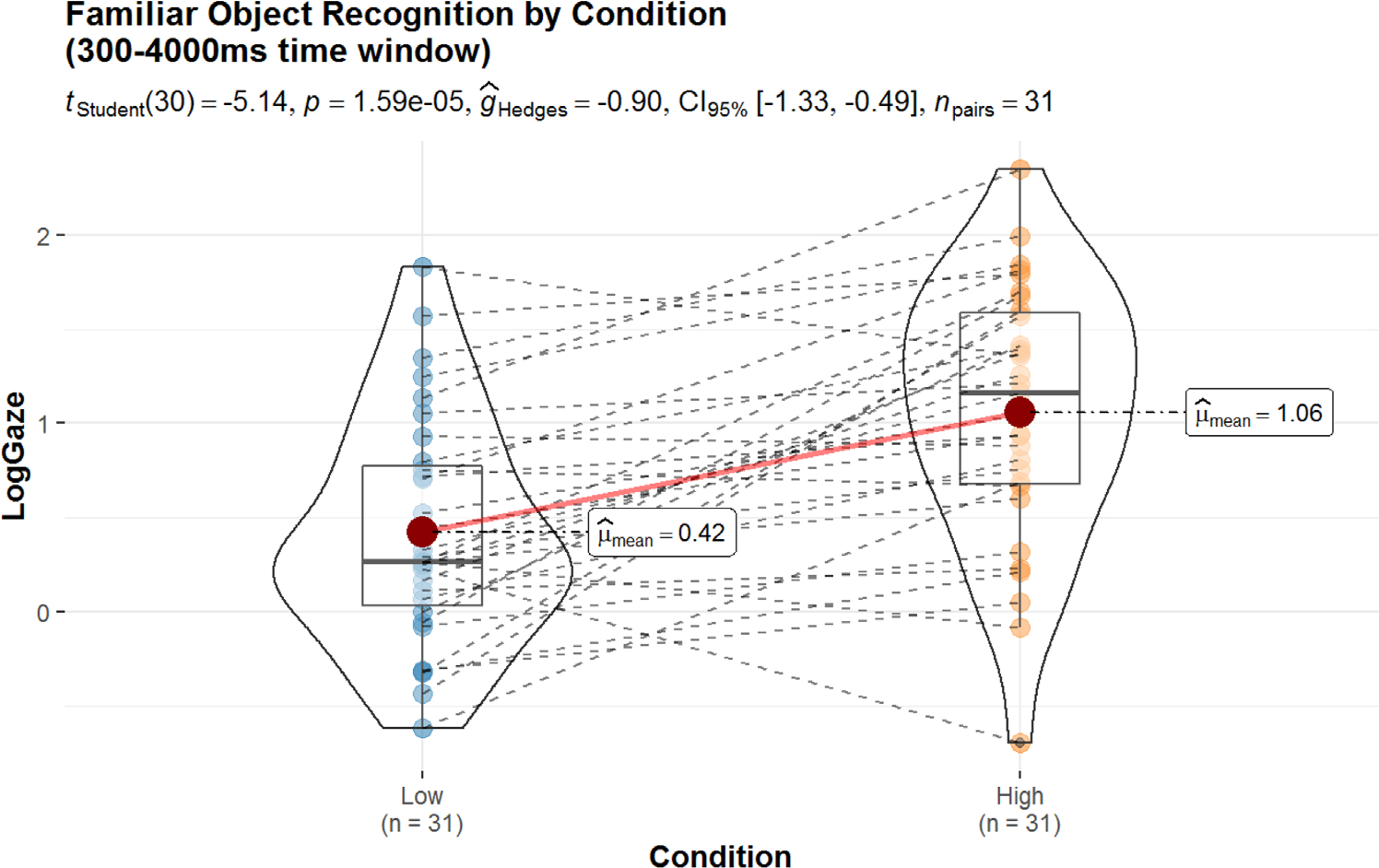
Differences in distribution of accuracy across High/Low conditions averaged across a 300–4000 ms time window. Violins illustrate the distribution of individual responses, while boxes indicate mean response in red and show first quartile, median, and third quartile of responses. Dotted lines show responses for individual participants across conditions. Log gaze responses greater than zero indicate a preference for the target across the time window, while negative values indicate a preference for the distractor.

Next, we statistically examined whether there were differences as a function of sensory condition using a time window accuracy analysis. Each time window was defined as the log-proportion of fixations to the Target versus Distractor across the entire trial period starting from 300 to 4000 ms post word onset. This window was selected for analysis to allow for consistent time window measurement between Experiments 2 and 3. While it is more typical in studies of familiar word processing to use a relatively shorter time window of analysis (300–1800 ms is typically recommend; Fernald et al., 2008), longer time windows are frequently employed when measuring recognition of novel words (see discussion in Bion et al., 2013; Borovsky, 2020). As in Bion et al. (2013), we selected the time period starting 300 ms post-word onset and spanning the entire trial window for both the current experiment focused on familiar words and the next experiment (Importantly, analysis with a more typical time window spanning 300–1800 ms, revealed identical statistical patterns). The distribution of log-gaze accuracy over the time window employed here, in relation to individual performance across each condition is illustrated in Figure 4. As shown, higher sensory words were recognized more accurately than lower sensory words (M_high_ = 1.06, M_low_ = 0.42, *t*(30) = −5.14, *p* < 0.0001), and this yielded a large effect (g_hedges_ = −0.90).

Next we explored whether and how age and vocabulary skill interacted with performance on each condition in this task using linear-mixed effects regression (LMER). These analyses were carried out using the lme4 library in R, version (Bates et al., 2015; R Core Team, 2019). Fixed effects of condition, vocabulary percentile and AoA were entered into the models. The condition factor was coded with High as the base level (High = 0, Low = 1), and AoA (in months) and vocabulary percentile were included in the model as centered and scaled variables, to facilitate interpretation of fixed effects estimates in the models. Random effects of Participants and Items were also included in the model. Model results are reported in Table 5. The statistical formula representing this statistical model was:

$$LogGaze\sim Condition\;*\;Vocabulary\;Percentile\;+\;AoA\;+\left(1\;\right|\;Subjects)\;+\;(1\;|\;Items).$$

This analysis revealed several effects. First, the positive intercept value indicated that, on average, children successfully recognized the labeled object by directing their gaze towards the target object during the analyzed time window. We also note that this intercept effect was stronger in the traditional familiar word time window analysis between 300 and 1800 ms (which is reported in the analytic code and results). The marginal (but positive) effect in the longer 300–4000 ms time window reflects that children showed less looking to the target at the end of the time window (illustrated in Figure 3). The significant effect of condition also aligns with the *t*-test comparison, with the positive estimate value indicating that higher sensory words were recognized more accurately than low sensory words. There were no other significant effects in this analysis, suggesting that these patterns were not driven by vocabulary skill or by item-level differences in (normative) AoA (Table 5).

In short, results are consistent with the findings from Experiment 1 and suggest that words with greater numbers of distinct senses linked with their meaning are more accurately recognized. This pattern may occur because having access to more senses might support the acquisition of robust representations. To further explore this hypothesis, in Experiment 3 we asked whether words are learned better when children have initial access to a greater number of distinct senses linked with their intended referents.

## 4 | EXPERIMENT 3

Using a within-subjects design, in Experiment 3 we explored whether different levels of multisensory exposure to an object impacts subsequent novel word learning. Thus, sensory exposure to two objects, prior to novel word learning, occurred as visual+tactile for one object and as visual-only for another object. This allowed us to directly test whether the number of sensory cues (and or the inclusion of touch as a sensory cue) during an exposure impacts later mapping to novel wordforms. As highlighted in the example earlier with applesauce, there are many sensory cues which we could have chosen to manipulate in this experiment (visual, auditory, tactile, gustatory, olfactory). While much past experimental work has focused on auditory and visual cues and shown that synchronous audio-visual cues can support word learning (e.g., Gogate, 2020; Gogate et al., 2009; Weatherhead et al., 2021; Yu & Smith, 2012), as a first step in examining how the number of distinct sensory cues might impact word learning we focus on the role that an additional tactile exposure might play in novel word learning for a few key reasons.

First, one only needs to observe infants for a brief period to realize that infants in the real world spend a lot of their time in tactile exploration with objects which teaches them about these objects (e.g., Wilcox et al., 2007) and maternal touch simultaneous with spoken words facilitates young infants’ learning of words for body parts (Tincoff et al., 2019). Second, given that the tactile system is the first to develop (and the visual system last), we expect that the tactile system may have a privileged place in the development of sensory integration and also in learning (Robinson & Sloutsky, 2010). Third, the perception of touch has long been appreciated as central to human perceptual development (e.g., Von Helmholtz, 1867) and is also a significant social signal in early development (Stack & Muir, 1990, 1992). We propose that examining how infants use touch perception in audition to sound and vision to help them to learn words can provide an ecologically valid understanding of underlying learning mechanisms and can reveal whether and how learning mechanisms scale to multisensory input. In sum, both within the broad context of perceptual development and the practical necessity to understand factors that contribute to language development, we cannot understand how children learn language until we understand the ways in which multisensory input, including touch, impacts acquisition.

### 4.1 | Methods

This experiment was identical in design to Experiment 2, except that we added a brief exposure phase and brief learning phase before testing. The exposure and learning phase occurred before Experiment 3′s test phase. The test phase was similar to Experiment 2. Exposure and learning phases were added so that we could explore how manipulation of the number of senses in an exposure phase would affect subsequent learning of a novel word.

#### 4.1.1 | Participants

Participants were the same as in Experiment 2.

### 4.2 | Materials/Experimental stimuli

#### 4.2.1 | Novel item selection

We selected two novel, physical objects which we expected to be unknown to participants: A red turkey baster bulb and a similarly sized off-white paint roller cover. Children’s lack of knowledge of these objects was further verified via the parental survey administered after the experiment and only children who were unfamiliar with these objects were included in the analyses (Appendix B).

#### 4.2.2 | Visual stimuli

Novel visual experimental stimuli consisted of photographs of the two yoked target objects (400 × 400 pixel color images), similar to Experiment 2. These images were placed on a green background (see Appendix A for photographs of each object used).

#### 4.2.3 | Auditory stimuli

Auditory stimuli were recorded at the same time, and using the same sampling rate and recording settings, as in Experiment 2. The two words recorded here were the novel words “toma” and “geeney.” These words were selected since both were bisyllabic novel words with trochaic stress, were distinct from each other, and contain phonemes that should be present in each child’s productive inventory by 26 months. In addition to recording the target words in isolation (as was done in Experiment 2), we also recorded the labels “toma” and “geeney” for an additional learning phase of the experiment described below.

#### 4.2.4 | Experimental procedure

The experimental procedure was similar to Experiment 2, except that it contained two additional phases: Exposure and Learning (which occurred before Experiment 3′s test phase).

##### Exposure.

**FIGURE 5:**
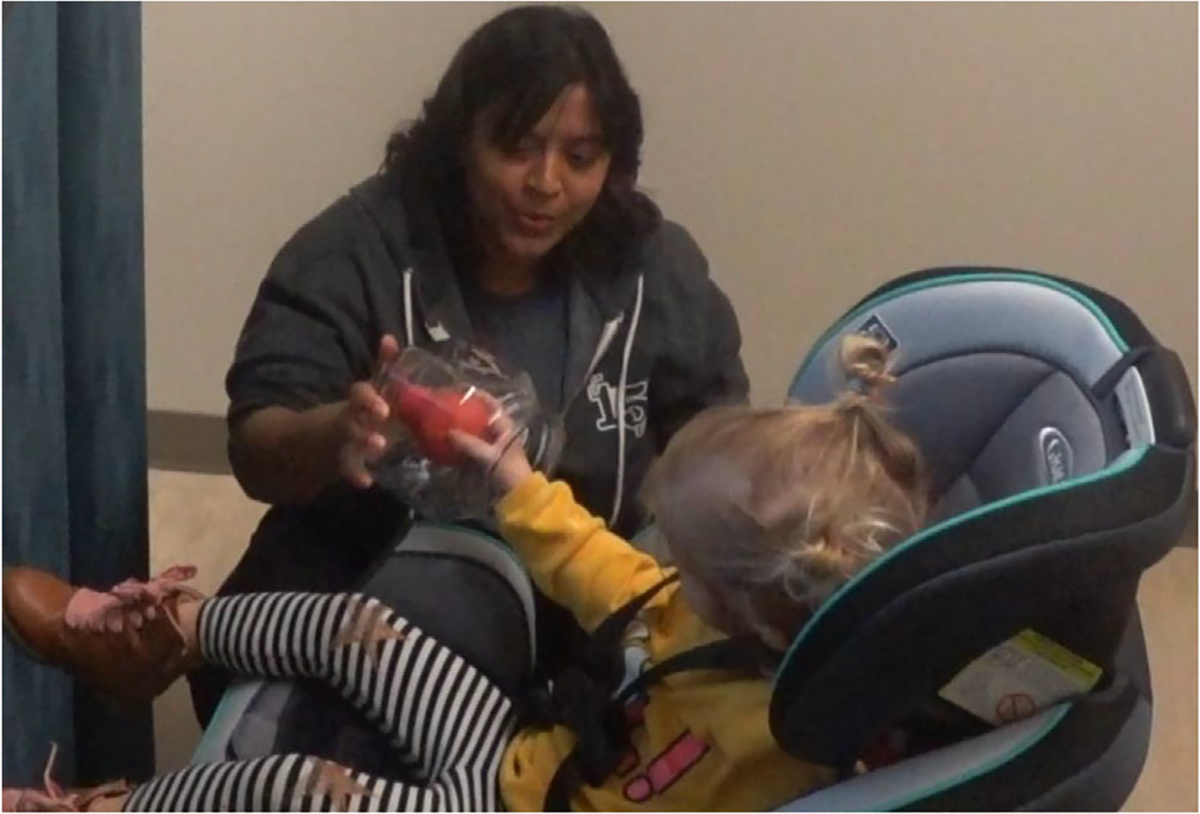
Tactile object exposure to one object, non-tactile exposure is identical except for the presence of a clear lid that prevented the object from being touched.

Post-calibration when the gray screen appeared on the monitor, an experimenter to the right of the participant instructed the participant that they would see an object and see-and-touch another object as part of a game. The experimenter then presented the child with two real novel objects for 10- to 12-seconds, one at a time in a counter-balanced order (Figure 5). Note that the baster bulb was the tactile-visual object for half of the children and a visual-only object for the other half of the children (and vice-versa for the paint roller). The experimenter controlled the time of exposure and the distance between the object and the child. Note that both tactile-visual and visual-only objects were held at the same distance from the child across conditions. The only difference between the two exposure conditions was that during the visual-tactile exposure, the child was allowed to touch the object. Specifically, the experimenter told infants, “I have two objects in this bag (bag hiding the objects) here. One, I’m going to let you touch, the other one, I’m not going to let you touch. This is part of my game!.” The experimenter only allowed tactile exploration of the “touch” object since a glass lid blocked exploration of the visual-only object.

##### Learning.

After this exposure phase, the two objects were shown on a screen, one at a time, in a Learning phase and named with an audio file as either “toma” or “geeney.” Specifically, children looked at the monitor and heard “Toma! There’s the toma!” repeated two times and “Geeney! There’s the geeney!” repeated two times. The presentation of the objects were counterbalanced across all exposure and learning phases.

##### Test.

After both the Exposure Phase and the Learning Phase the child entered the Test Phase. During the Test Phase, as in Experiment 2, infants looked at the two objects side-by-side on the screen and heard utterances like “Look! Geeney/Toma!” followed by an encouragement phrase (e.g., “Yeah! That’s it! or ”Great job!”). Similar to Experiment 2, after the experiment, each caregiver was asked to rate their child’s knowledge of the two novel items on a scale from 1 (“child does not say/understand the word”) to 4 (“child says/understands the word”).

#### 4.2.5 | Data cleaning

As mentioned, all trials were removed if caregivers indicated any knowledge of novel objects (“2”, “3,” or “4” on our questionnaire; e.g., knowing the turkey baster prior to testing resulted in removal of all trials). Caregivers indicated that one participant understood (a rating of a 3 out of 4 meaning that they did understand or say the word for the item). This participant’s data were removed from the analysis in this experiment, leaving 30 participants in the experiment. Otherwise, participants’ caregivers rated the child’s knowledge of each item as a 1 or 2 out of 4—that is their children didn’t know either the “bulb” or the “roller.”

The 30 participants in the remaining dataset completed 232 trials (See: 117; Touch: 115). As in Experiment 2, trials were removed for excessive track loss (defined as less than 20% of the samples in the trial period available). With this criterion an additional 11 trials (4.7% of trials) were removed from the analysis. After track loss removal, there were 221 trials remaining (See: 111 trials; Touch: 110 trials) that were submitted to the final analysis.

### 4.3 | Results

#### 4.3.1 | Visualizing novel word recognition

**FIGURE 6:**
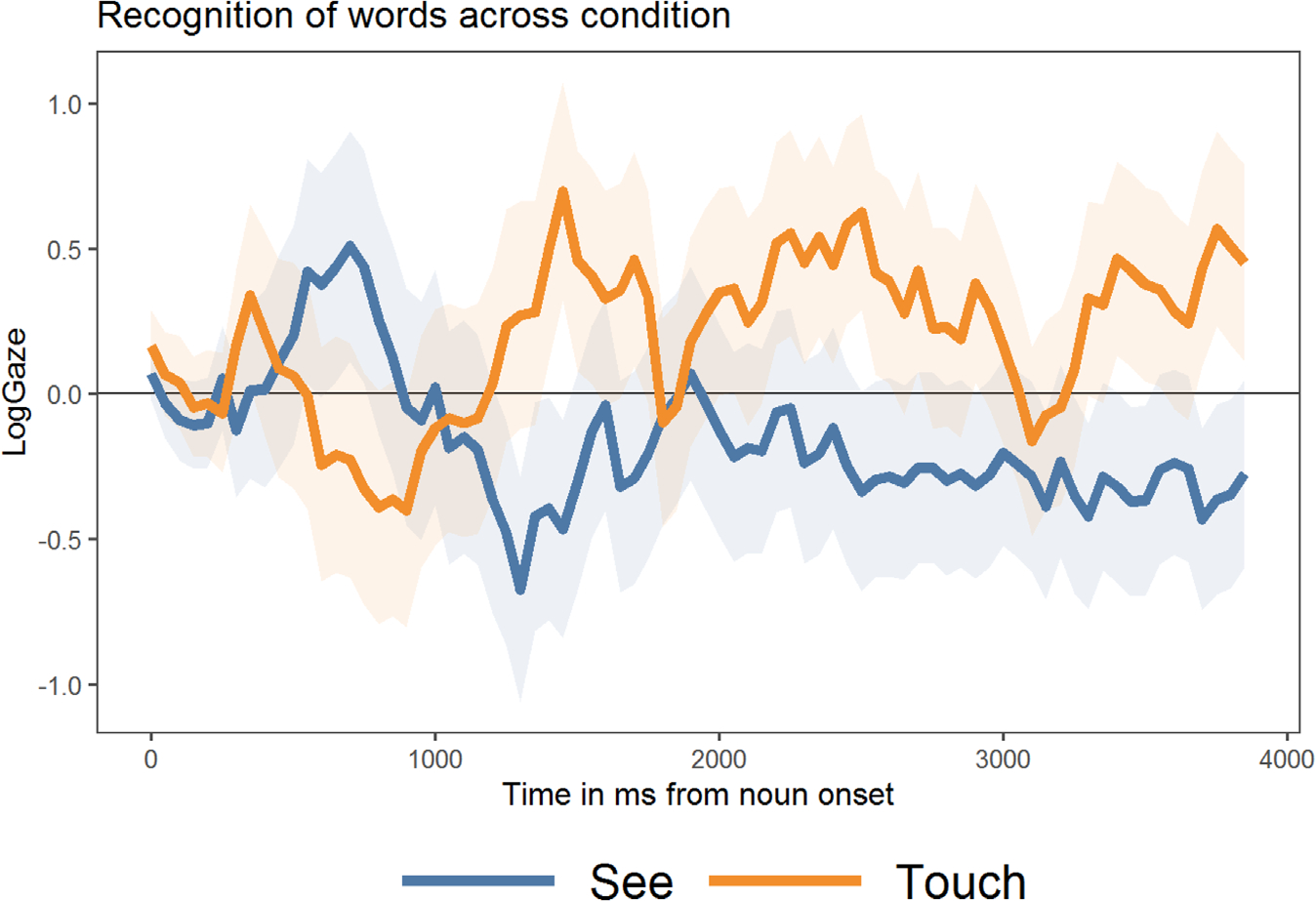
Timecourse of novel word recognition across See and Touch words plotted in 50 ms time bins. Positive values indicate a target preference, and negative values indicate a preference to look at the distractor image.

We first visualized the time course of recognition of novel words in the two experimental conditions (1) See-only (See) and (2) See+Touch (Touch) in a timecourse plot (Figure 6) as we did for Experiment 2. There are several apparent patterns in this plot. First, looks towards the target object, when compared to familiar word recognition trials, did not appear to be as robust. Rather than quickly following a rapid positive slope following word onset, children’s gaze pattern exhibited a pattern that indicated no preference for the target or distractor object for the first 2000 ms following label onset, across both experimental conditions. After 2000 ms, however, children in the Touch condition showed, on average, a target preference (indicated by positive log gaze values across time), until the end of the trial period at 4000 ms post word onset. This rise in positive LogGaze fixation proportions within the first 500–1000 ms after spoken word onset indicates that, as expected, toddlers recognized the spoken labels and directed their gaze towards the appropriate target image. This plot also illustrates a clear difference in the timing—such that it appeared to take longer for toddlers to direct fixations towards the target objects in the lower sensory condition (See), and that this pattern persisted through much of the plotted time period. In the next section, we explored these visually apparent patterns through statistical analysis.

#### 4.3.2 | Time window accuracy analysis

**FIGURE 7:**
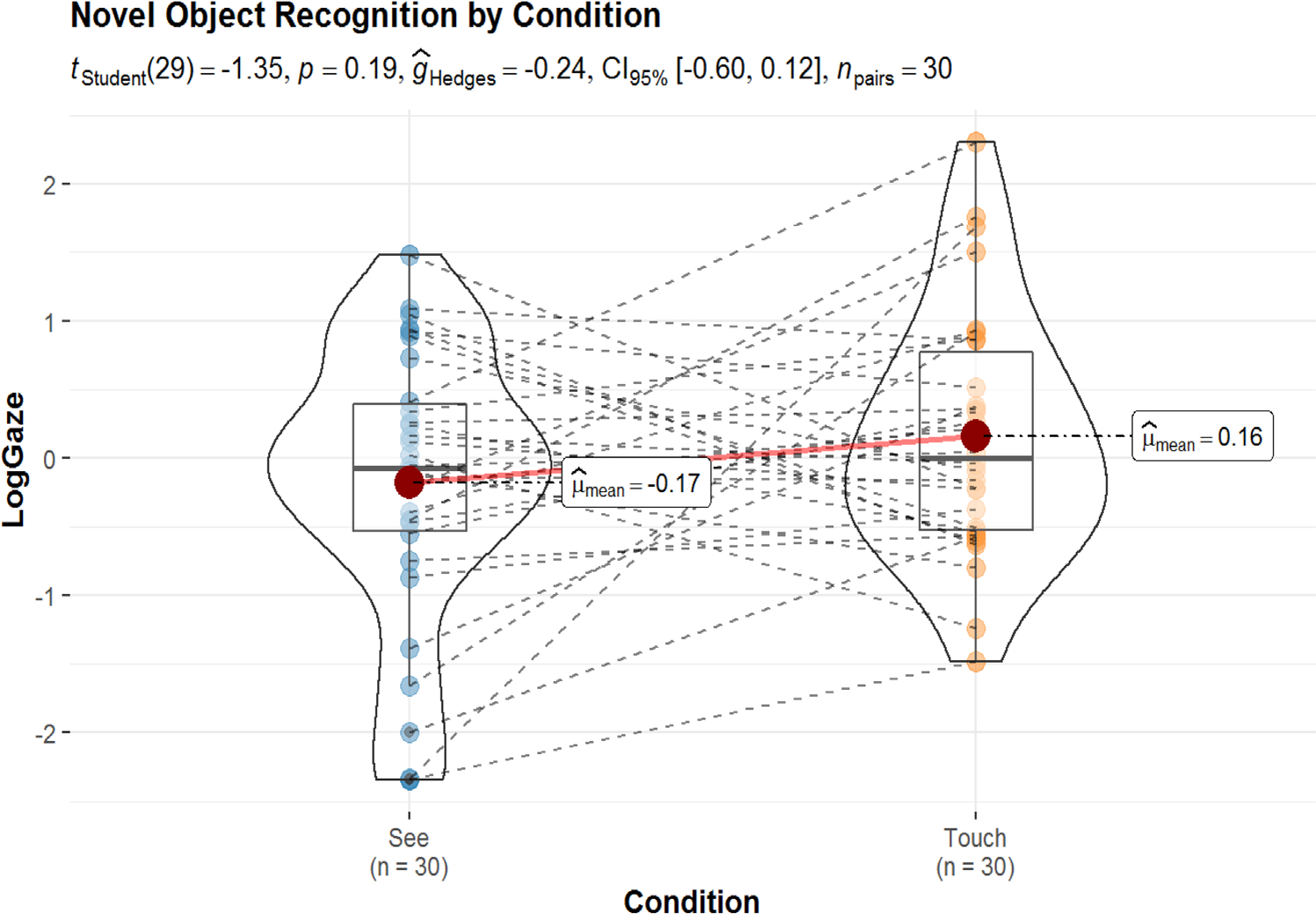
Mean log gaze fixations from 300 to 4000 ms post novel label onset.

We employed the same analytic approach as in Experiment 2, except for the inclusion of AoA in our LMER model, as item-level AoA values do not exist for the (constructed) novel items presented in this study. Like in Experiment 2, we averaged log-gaze across a time window spanning 300–4000 ms. We then compared average looking in this time window across the See and Touch conditions using paired t-tests (illustrated in Figure 7). This comparison did not reach significance (*p* = 0.19) and the measure of effect size (g_hedges_ = −0.24) indicated a small difference across conditions. Next, we sought to explore whether vocabulary skill influenced performance on this task using a LMER modeling approach that mirrored the approach in Experiment 2. Again, fixed effects of condition and vocabulary percentile were entered into the model. Condition was entered as a factor with the See condition as the base level (0 = See, 1 = Touch), and vocabulary percentile was entered as a centered and scaled variable to facilitate comparison across effects. The formula for this analysis was:

$$\begin{array}{rr} & \text{LogGaze}\sim \text{Condition}*\text{VocabularyPercentile}+\text{AoA}\\ & \;\;\;\;\;\;+(1|\text{Subjects})+(1|\text{Items}). \end{array}$$

The results of the LMER analysis are reported in Table 6. This analysis revealed a significant effect of Condition, with more robust recognition of words in the Touch condition relative to the See condition. No other factors or interactions were significant in the model.

## 5 | GENERAL DISCUSSION

In a series of three experiments using a converging methods approach, we asked whether and how the acquisition and understanding of words may be influenced by the number of sensory experiences associated with linked objects. We considered two possibilities: more multisensory experiences with objects linked to words could either support or hinder word learning and recognition. The cross-study results are clear: a greater number of sensory experiences support early lexical acquisition and recognition. Experiment 1 demonstrated that children acquire words earlier in development when they are linked with objects that have a greater number of associated sensory experiences. In Experiment 2 toddlers’ recognition of words was facilitated for words that have more senses linked with their meaning. Finally, although this experiment only showed a small effect size in the difference between conditions, in Experiment 3 toddlers’ word learning was supported when their initial exposure to a novel object included both tactile and visual access, compared to visual-only access.

What are the mechanisms that drive this multisensory lexical boost across these three experiments? Along with previous studies which find that multisensory information supports word learning (e.g., Gogate et al., 2000), this data supports the theory that multisensory information may guide word learning (e.g., the Multisensory Underpinnings of Lexical Comprehension hypothesis; Gogate & Hollich, 2010, 2016). We thus posit that word-form to word-meaning mappings can be supported by expanding the type of learners’ experiences with objects via multiple sensory channels and highlight two potential pathways through which multisensory information linked to objects could support word learning and word recognition (note that these two pathways are not mutually exclusive).

First, it is possible that a greater number of multisensory properties associated with objects serves to increase the semantic specification linked to a lexical form. In other words, objects with a greater number of sensory cues linked with them (e.g., *banana* has smell, taste, visual, and touch affordances (*n* = 4) while *sky* only has visual (*n* = 1)), benefit from richer representations, which serves to support acquisition and processing. This lexical strengthening due to semantic specification idea is consistent with prior results showing a supportive role in word learning with semantic specification (Heisler et al., 2010). For example, in Heisler et al. (2010) words which were associated with more semantic information/depth were learned better and articulated more clearly for both typical and atypical language learners. The specific pathway proposed here would additionally build on this account by highlighting another pathway—sensory affordances—that can enrich lexical representations for words associated with objects.

The findings from the novel word learning study in Experiment 3 also suggests that this multisensory boost in learning is tied to direct experience with information in multiple sensory domains, as opposed to sensory experience that might be “inferred” or “simulated” through visual observation alone. For example, the tactile properties of the novel objects in Experiment 3 could have potentially been inferred through visually-apparent differences in texture. Nonetheless, our findings indicate that experience with physically touching the object supported subsequent mapping and retention of a lexical label over visual experience alone. More broadly, this pattern suggests that children who experience differences in sensory experience (i.e., as in children who are blind, deaf, or have sensory sensitivities) may seek out or prioritize direct sensory experiences in other channels to support their language learning. At the same time, this “direct sensory experience” mechanism should not be subject to cultural variation given that the physical and sensory features of objects do not change with culture, that is, a banana has the same affordances in Tanzania and Italy. Thus, while this pathway would not predict cultural variation, it would predict child-level variation. We term this pathway the *semantic enrichment pathway* since it suggests that multisensory exposure enhances learning by expanding and strengthening the network activation associated with the lexical representation of the word.

Another possible (not mutually exclusive) pathway is the idea that multisensory properties of objects enhance or create more learning opportunities for the child which, in turn, supports their mapping and retention. These learning opportunities can be driven by the caregiver and/or the child. For example, if an object has a salient visual, olfactory, and gustatory feature (e.g., banana), caregivers may highlight these senses when the object is attended to by the child (Schroer & Yu, 2022). Alternatively (or in addition), the child’s own interests may guide their word learning (Ackerman et al., 2020), such that the child’s own curiosity may drive sensory exploration, which may, in turn, facilitate label learning. In contrast, items with fewer senses linked with their meaning may lead to fewer opportunities for caregiver input and child exploration. Further, unlike the semantic enrichment pathway, this pathway may be subject to both child-level variation (as a function of individual interest and exploration) and cultural variation. Specifically, caregivers in different cultures may be more or less likely to discuss/focus different sensory cues which may be more or less culturally and linguistically salient. For example, with respect to linguistic variation, Gogate et al. (2000) and Gogate et al. (2015) show that, although caregivers across cultures both readily exploit multimodal input synchrony, there are language-specific differences in the amount of maternal auditory-visual-tactile/auditory-visual behaviors in Indian and American caregivers for nouns and verbs which may be driven by linguistic structural differences. Similarly, linguistic and cultural information can interact in ways that impact multisensory input. For example, languages vary significantly in the degree to which information about sensory features are expressed: olfactory features are especially variable, such that in some languages (such as Jahai) color and odor naming skills are equivalent while in other languages (e.g., English) they are not (Majid & Burenhult, 2014). We term this the *learning opportunities pathway* since it suggests that objects with more multisensory affordances (or more culturally prioritized affordances) would generate a greater number of *learning opportunities/attention* to objects and this would contribute to the strength of the lexical representation of new and known words.

The learning opportunities pathway is additionally supported by a body of work highlighting that the frequency of wordforms and hence, of learning opportunities, provided by caregivers for children impacts learning (e.g., Swingley & Humphrey, 2018), however, raw frequency alone does not account for acquisition of words as well as more complex models that include the child’s sensory experiences (as illustrated in the models in Experiment 1; Abu Zhaya et al., 2017; Amatuni et al., 2021). Thus, it is likely that objects linked with more senses might induce more learning opportunities since caregivers are likely to talk about those senses (e.g., a caregiver would be more likely to highlight the smell and taste of banana, but not the smell/taste of the sky), but that these learning opportunities alone do not account wholly for when words are acquired (e.g., Clerkin & Smith, 2022). This broad principle is also supported by recent data suggesting that frequency and learning opportunities (Yu & Smith, 2012) impact word learning and other work from this lab suggest that visual referents do not serve well to explain why words are acquired when they are (Clerkin & Smith, 2022). For example, Sun and Yoshida (2022) suggest that caregivers’ attention and naming use induces optimal learning opportunities that learners must capitalize on. In short, this pathway suggests that these opportunities may be likely to occur more frequently with objects with multisensory features and that, in turn, impacts the robustness of the child’s representation. More work is needed to explore how these sensory aspects of word meanings are highlighted in child-directed speech to directly assess this idea.

The learning opportunities pathway predicts that the amount of exposure to these words and the focus on sensory cues in the input will vary with culture, SES, and individual caregivers since such behaviors vary with culture (e.g., Richman et al., 1988; Roopnarine et al., 2005) and that such variations in exposure and emphasis will impact infant learning behavior and, indirectly, sensitivity to such cues (Wefers et al., 2023). For example, Richman et al. (1988) shows that infants growing up amongst Kenyan Gusii caregivers or Mayan caregivers get held and touched more than two times as much as those growing up in the US, Italy, or Sweden, but get talked to half as much as those growing up in Sweden. These differences in caregiver behavior seem to impact infant sensitivity to cues with infants growing up in high touch environments appearing to be more sensitive to variations in touch and the withholding of this cue (e.g., Wefers et al., 2023). Similarly, child-directed visual exposure with and without touch varies with culture, with some cultures using more touch when exposing infants to visual objects vs. others. For example, infants in Vanuatu experience more *physical* triadic engagement with novel objects, while those in the US experience more *visual* triadic engagement (Little et al., 2016). Like touch, approaches to olfaction also vary across cultures. English-speaking cultures focus little on olfaction, but olfaction is discussed more frequently in many other cultures (Majid, 2021). Finally, the amount of caregiver speech and quality of caregiver speech varies with culture and SES. For example, recent work suggests that infants learning Tsimane or Mayan may hear less than 50% of the amount of infant-directed input than infants growing up in the US (Cristia et al., 2019; Shneidman & Goldin-Meadow, 2012). Given the range of cultural variation in multisensory exposure in interacting with infants, it is important to understand that multisensory exposure and focus may differentially impact infant word learning opportunities. Thus, a clear limitation in this work is that we did not explore which sensory cues might be most helpful in word recognition and word learning. Future work will need to explore whether certain sensory cues may be more helpful than others. Nonetheless, in this paper, we took a first pass at addressing this question by exploring the impact of multisensory exposure on word learning and AoA in one culture.

## Supplementary Material

Supplementary data Exp1

supplementary data Exp2+3

## Figures and Tables

**TABLE 1:** Example of semantic and perceptual feature classification and sensory modality measurement for a single concept, “Apple.”

Features	Semantic feature category	Perceptual feature
is_red	Perceptual	Visual
a_fruit	Taxonomic	
grows_on_trees	Encyclopaedic	
is_green	Perceptual	Visual
eaten_in_pies	Function	
is_crunchy	Perceptual	Tactile
has_seeds	Perceptual	Visual
is_juicy	Perceptual	Tactile
tastes_sweet	Perceptual	Taste
is_round	Perceptual	Visual
is_delicious	Perceptual	Taste
is_nutritious	Encyclopaedic	
is_yellow	Perceptual	Visual
has_a_core	Perceptual	Visual
has_skin	Perceptual	Visual
tastes_sour	Perceptual	Taste
used_for_cider	Function	
eg_-_granny_smith	Taxonomic	
is_worm_infested	Encyclopaedic	
**Number of features: 19**	**Number of perceptual features: 12**	**Number of distinct senses: 3**

**TABLE 2:** Effects of number of distinct senses on AoA, while controlling for frequency, concreteness, and overall number of features.

	Estimate	Std. Error	t-statistic	p-value
ADS frequency	0.04	0.28	0.14	0.89
CDS frequency	−4.26	0.32	−13.18	<0.0001
Concreteness	−0.58	0.65	−0.89	0.37
N of features	−0.07	0.05	−1.40 to −1.39	0.16
Distinct senses	−0.62	0.22	−2.8	0.005
Constant	36.9	3.06	12.05	<0.0001
*R* ^2^_adj_	= 0.46			
F (5,330)	= 59.1	*p* < 0.0001		

*Note* estimates are reported in *β* (not *β*_std_) to facilitate interpretation of relation between senses and AoA. These statistical patterns are identical when variables are standardized, and reported in analytic code.

**TABLE 3:** Effect of number of senses on age of acquisition, while controlling for frequency, concreteness and number of perceptual features (NoPF).

	Estimate	Std. Error	t-statistic	*p*-value
ADS frequency	0.05	0.28	0.19	0.85
CDS frequency	−4.26	0.32	−13.21	<0.0001
Concreteness	−0.46	0.67	−0.69	0.49
NoPF	−0.08	0.06	−1.40	0.16
Distinct senses	−0.61	0.22	−2.73	0.007
Constant	35.8	3.18	11.25	<0.0001
*R* ^2^_adj_	= 0.46			
*F* (5,330)	= 59.2	*p* < 0.0001		

*Note* estimates are reported in *β* (not *β*_std_) to facilitate interpretation of relation between senses on AoA. These statistical patterns are identical when variables are standardized, and reported in analytic code.

**TABLE 4:** Participant demographics and related measures for Experiments 2 & 3.

Participants	Summary data
*Age*	M = 26.6 (R = 24.14–30.53)
*Sex*	12 M; 19 F
*% White*	83.9%
*% on MBCDI:WS*	M = 50.4% (SD = 28.9)
*Percentage mothers completed college*	93.5%

**TABLE 5:** Linear mixed-effects model in Experiment 2.

	LogGaze		
*Predictors*	*Estimates*	*CI*	*p*
(Intercept)	0.43	−0.03 to 0.89	0.068
Condition [High]	0.65	0.02 to 1.27	**0.042**
Percentile	0.10	−0.13 to 0.33	0.395
AoA	−0.01	−0.32 to 0.29	0.933
condition [High] * percentile	−0.15	−0.41 to 0.11	0.260
**Random effects**			
*σ* ^2^	2.87		
*τ*_00_ Subject	0.17		
*τ*_00_ Item	0.24		
ICC	0.12		
N _Subject_	31		
N _Item_	12		
Observations	672		
Marginal R^2^ / Conditional R^2^	0.033/0.154		

*Note:* Analyses was carried out on log-gaze looking over time window spanning 300–4000 ms.

The bold value is statistically significant at the traditional *p* > 0.05 level.

**TABLE 6:** Linear mixed effects modeling results of Experiment 3.

	LogGaze		
*Predictors*	*Estimates*	*CI*	*p*
(Intercept)	−0.19	−0.86 to 0.47	0.568
Condition [Touch]	0.43	0.05 to 0.81	**0.028**
Percentile	0.06	−0.23 to 0.36	0.684
Condition [Touch] * percentile	0.19	−0.20 to 0.58	0.334
**Random effects**			
*σ* ^2^	2.09		
*τ*_00_ Subject	0.09		
*τ*_00_ Item	0.18		
ICC	0.12		
N _Subject_	30		
N _Item_	2		
Observations	221		
Marginal R^2^/Conditional R^2^	0.033/0.144		

**Table:** Yoked stimuli for each experiment with words, number of senses, AoA, and photos used.

Experiment	High word and number of senses	Mean AoA for High (months)	Low word and number of senses	Mean AoA for Low (months)	Visual display for High and Low words
2	Hammer (3)	25.24	Pencil (1)	24.60	
2	Balloon (3)	16.84	Socks (1)	20.17	
2	Candy (3)	22.01	Button (1)	21.79	
2	Apple (3)	17.79	Finger (1)	21.31	
2	Pig (3)	20.33	Truck (1)	18.56	
2	Doggy (3)	13.02	Car (1)	16.93	
3	Either bulb or roller, depending on condition		Either bulb or roller, depending on condition		

Novel word and object forms.

## Data Availability

The deidentified / blinded data analyzed for this paper and analysis scripts are available here: (https://osf.io/7q29e/?view_only=16aee6741a93495bba1170680edc00e4).

## Research Highlights

- Infants learn words in rich multisensory environments, but some words are linked with more senses than others (e.g., banana is linked with more senses than sky).
- In three experiments, we examined whether the number of senses linked with objects impacts learning of words linked with objects.
- Converging evidence across all three experiments indicated that words linked with objects that triggered more senses were learned earlier and better.

## Footnotes

1 At the suggestion of a reviewer, we also carried out a parallel set of analyses with these two items removed from the dataset as well. The patterns are identical in both approaches, and the analysis with the items removed is included in the analytic code for additional reference.

2 Note also that the term “number of features” (NoF) is a measure that has been previously used in the literature which includes a tally of the raw number of semantic features (across all feature categories. It has been posited that number of features reflects semantic richness.
