# Supplementary material for: Touch to learn: Multisensory input supports word learning and processing
Source: Dev Sci. Author manuscript; Available in PMC 2024 Feb 21. (PMC10704002; doi:10.1111/desc.13419)
Supplement: Supplementary data Exp1 [file NIHMS1926655-supplement-Supplementary_data_Exp1.zip › Exp1_AoA_Analysis_Final.html]

Exp1\_Analysis


# Exp1\_Analysis

#### Arielle Borovsky

- Statistical Analysis
- Post-hoc,
  supplemental statistical analyses with mommy and daddy items
  removed

Analyses for Experiment 1 of “Touch to Learn” manuscript. Exploring
relation between Age-of-Acquisition and number / type of sensory
modalities Revision 1, Developmental Science.

## Statistical Analysis

Explore relation between AoA and triggered senses

```
#Is AoA predicted by Number of Distinct Senses associated with semantic features of a word,  when controlling for  frequency (Adults + CDS), Concreteness, and overall Number of Semantic Features associated with a word?  

m1 <- lm(Wordbank_aoa ~ 
           log10(SUBTLWF) + 
           log10(CHILDES.freq.pm) + 
           Conc.M + 
           Num_Feats_All + 
           DistinctSenses,
          data=SLTL_AoA_CDINouns)
summary(m1)
```

```
## 
## Call:
## lm(formula = Wordbank_aoa ~ log10(SUBTLWF) + log10(CHILDES.freq.pm) + 
##     Conc.M + Num_Feats_All + DistinctSenses, data = SLTL_AoA_CDINouns)
## 
## Residuals:
##      Min       1Q   Median       3Q      Max 
## -10.5071  -1.3655   0.1509   1.5107   9.5293 
## 
## Coefficients:
##                        Estimate Std. Error t value Pr(>|t|)    
## (Intercept)            36.86764    3.05929  12.051  < 2e-16 ***
## log10(SUBTLWF)          0.03815    0.27902   0.137  0.89134    
## log10(CHILDES.freq.pm) -4.25780    0.32297 -13.183  < 2e-16 ***
## Conc.M                 -0.58264    0.65283  -0.892  0.37279    
## Num_Feats_All          -0.06785    0.04869  -1.394  0.16439    
## DistinctSenses         -0.62462    0.21884  -2.854  0.00459 ** 
## ---
## Signif. codes:  0 '***' 0.001 '**' 0.01 '*' 0.05 '.' 0.1 ' ' 1
## 
## Residual standard error: 2.753 on 330 degrees of freedom
##   (23 observations deleted due to missingness)
## Multiple R-squared:  0.4726, Adjusted R-squared:  0.4646 
## F-statistic: 59.14 on 5 and 330 DF,  p-value: < 2.2e-16
```

```
#yes distinct senses is associated with AoA when controlling for overall frequency,  concreteness and number of features. 

#Next: What about if instead of overall Number of features,  we consider just overall number of perceptual features?   I.e. - is this simply a "perceptual semantic richness" effect?   

m2 <- lm(Wordbank_aoa ~ 
           log10(SUBTLWF) + 
           log10(CHILDES.freq.pm) + 
           Conc.M + 
           Num_Percep + 
           DistinctSenses,
          data=SLTL_AoA_CDINouns)
summary(m2)
```

```
## 
## Call:
## lm(formula = Wordbank_aoa ~ log10(SUBTLWF) + log10(CHILDES.freq.pm) + 
##     Conc.M + Num_Percep + DistinctSenses, data = SLTL_AoA_CDINouns)
## 
## Residuals:
##      Min       1Q   Median       3Q      Max 
## -10.8793  -1.3728   0.1981   1.5404   9.4634 
## 
## Coefficients:
##                        Estimate Std. Error t value Pr(>|t|)    
## (Intercept)            35.81629    3.18419  11.248  < 2e-16 ***
## log10(SUBTLWF)          0.05317    0.27828   0.191  0.84859    
## log10(CHILDES.freq.pm) -4.26076    0.32252 -13.211  < 2e-16 ***
## Conc.M                 -0.46226    0.67441  -0.685  0.49356    
## Num_Percep             -0.08196    0.05870  -1.396  0.16352    
## DistinctSenses         -0.60757    0.22254  -2.730  0.00667 ** 
## ---
## Signif. codes:  0 '***' 0.001 '**' 0.01 '*' 0.05 '.' 0.1 ' ' 1
## 
## Residual standard error: 2.753 on 330 degrees of freedom
##   (23 observations deleted due to missingness)
## Multiple R-squared:  0.4726, Adjusted R-squared:  0.4646 
## F-statistic: 59.15 on 5 and 330 DF,  p-value: < 2.2e-16
```

```
#number of distinct senses still assocaiated with AoA even after controlling for overall number of perceptual features
```

## Post-hoc, supplemental statistical analyses with mommy and daddy items removed

A reviewer suggested we remove these two items rather than replace
them with a low AoA due to their modeled negative AoA values - we re-run
the same analyses above with those items removed to verify that the
patterns are similar and share as supplemental analysis.

```
#first remove mommy and daddy from the dataset:

SLTL_AoA_CDINouns_noMomDad <- SLTL_AoA_CDINouns %>%
  filter(!Concept %in% c("mommy", "daddy"))


# Is AoA predicted by Number of Distinct Senses associated with semantic features of a word,  when controlling for  frequency (Adults + CDS), Concreteness, and overall Number of Semantic Features associated with a word?  

m1_noMomDad <- lm(Wordbank_aoa ~ 
           log10(SUBTLWF) + 
           log10(CHILDES.freq.pm) + 
           Conc.M + 
           Num_Feats_All + 
           DistinctSenses,
          data=SLTL_AoA_CDINouns_noMomDad)
summary(m1_noMomDad)
```

```
## 
## Call:
## lm(formula = Wordbank_aoa ~ log10(SUBTLWF) + log10(CHILDES.freq.pm) + 
##     Conc.M + Num_Feats_All + DistinctSenses, data = SLTL_AoA_CDINouns_noMomDad)
## 
## Residuals:
##     Min      1Q  Median      3Q     Max 
## -9.3798 -1.4082  0.1045  1.3835  9.2426 
## 
## Coefficients:
##                        Estimate Std. Error t value Pr(>|t|)    
## (Intercept)            39.49996    2.96125  13.339  < 2e-16 ***
## log10(SUBTLWF)         -0.02271    0.26702  -0.085 0.932260    
## log10(CHILDES.freq.pm) -3.93684    0.31391 -12.541  < 2e-16 ***
## Conc.M                 -1.20733    0.63364  -1.905 0.057603 .  
## Num_Feats_All          -0.06364    0.04657  -1.366 0.172754    
## DistinctSenses         -0.74593    0.21025  -3.548 0.000445 ***
## ---
## Signif. codes:  0 '***' 0.001 '**' 0.01 '*' 0.05 '.' 0.1 ' ' 1
## 
## Residual standard error: 2.632 on 328 degrees of freedom
##   (23 observations deleted due to missingness)
## Multiple R-squared:  0.4691, Adjusted R-squared:  0.461 
## F-statistic: 57.97 on 5 and 328 DF,  p-value: < 2.2e-16
```

```
#yes distinct senses is associated with AoA when controlling for overall frequency,  concreteness and number of features. 

#Next  What about if instead of overall Number of features,  we consider just overall number of perceptual features?   I.e. - is this simply a "perceptual semantic richness" effect?   

m2_noMomDad <- lm(Wordbank_aoa ~ 
           log10(SUBTLWF) + 
           log10(CHILDES.freq.pm) + 
           Conc.M + 
           Num_Percep + 
           DistinctSenses,
          data=SLTL_AoA_CDINouns_noMomDad)
summary(m2_noMomDad)
```

```
## 
## Call:
## lm(formula = Wordbank_aoa ~ log10(SUBTLWF) + log10(CHILDES.freq.pm) + 
##     Conc.M + Num_Percep + DistinctSenses, data = SLTL_AoA_CDINouns_noMomDad)
## 
## Residuals:
##     Min      1Q  Median      3Q     Max 
## -9.5124 -1.4502  0.1863  1.4782  8.8647 
## 
## Coefficients:
##                        Estimate Std. Error t value Pr(>|t|)    
## (Intercept)            38.08239    3.06011  12.445  < 2e-16 ***
## log10(SUBTLWF)         -0.01812    0.26555  -0.068  0.94564    
## log10(CHILDES.freq.pm) -3.89385    0.31363 -12.415  < 2e-16 ***
## Conc.M                 -0.99330    0.64933  -1.530  0.12705    
## Num_Percep             -0.11047    0.05614  -1.968  0.04993 *  
## DistinctSenses         -0.69156    0.21254  -3.254  0.00126 ** 
## ---
## Signif. codes:  0 '***' 0.001 '**' 0.01 '*' 0.05 '.' 0.1 ' ' 1
## 
## Residual standard error: 2.624 on 328 degrees of freedom
##   (23 observations deleted due to missingness)
## Multiple R-squared:  0.4723, Adjusted R-squared:  0.4643 
## F-statistic: 58.72 on 5 and 328 DF,  p-value: < 2.2e-16
```
