# Supplementary material for: Touch to learn: Multisensory input supports word learning and processing
Source: Dev Sci. Author manuscript; Available in PMC 2024 Feb 21. (PMC10704002; doi:10.1111/desc.13419)
Supplement: supplementary data Exp2+3 [file NIHMS1926655-supplement-supplementary_data_Exp2_3.zip › Exp2-3_Analysis_Final.html]

Experiment 2 & 3 Analysis


# Experiment 2 & 3 Analysis

#### Arielle Borovsky

#### 31 May, 2023

- Load
  Exp 2 and 3 data
- Experiment
  2: Familiar Trials – single word lexical recognition trials
  - Experiment 2: Timecourse
    plots
  - Experiment2:
    Binning data into aggregated windows:
  - View data across conditions
    - Compare in 300-1800 ms time
      window
    - Compare in 300-4000 ms time
      window
  - Statistical models
    - modelling
      performance in 300- 1800 time window
    - Models for
      recognition in 300-4000 ms time window
- Novel word analysis (
  Experiment 3)
  - Experiment 3: Timecourse
    plots
  - Aggregate Novel word into
    time bins
  - View data across conditions
    - Compare first in 300-1800
      ms window
    - Compare
      novel word recognition in 300-4000 ms time window
  - Statistical models
    - Comparing in 300-4000 ms
      window
  - SAVE

This script carries out the analysis for Experiments 2 and 3.

## Load Exp 2 and 3 data

```
load("../Output/Exp2Data.Rdata")
load("../Output/Exp3Data.Rdata")
```

# Experiment 2: Familiar Trials – single word lexical recognition trials

The goal of these trials is to measure how accuracy of single word
recognition varies according to the number of sensory modalities
associated with the semantic features of the word

## Experiment 2: Timecourse plots

visualize the timecourse

```
#FF - Exp 2

FFPlt<-ggplot(FF_ET, aes(x=Time, y=LogGaze, group=condition, color=condition, fill = condition)) +
  labs(title = "Recognition of words across condition", x="Time in ms from noun onset") +
  geom_hline(yintercept = 0)+
  stat_summary(fun.data=mean_se, geom="ribbon", alpha=.2,color=NA,show.legend = FALSE)+
  stat_summary(fun=mean, geom="line", size=2) + 
  scale_color_tableau()+
  scale_fill_tableau()+
  theme_few()+
  theme(legend.text=element_text(size=18),
        legend.title=element_blank(),
        legend.position="bottom") +
  guides(linetype=guide_legend(keywidth = 5, keyheight= 1),
         color=guide_legend(keywidth= 5, keyheight = 1))
FFPlt
```

```
ggsave("../Figs/FF_TC.png")
```

```
## Saving 7 x 5 in image
```

## Experiment2: Binning data into aggregated windows:

As a first step, we bin the data into large time windows. Eventually,
we should do GAMs for timecourse measurement, which incorporates
differences in timing. We plot LogGaze across each time window.

```
FF_Agg <- FF_ET %>% 
  dplyr::filter (Time > 300 & Time < 1800) %>%                  #these numbers define your time window - we're using a standard 300-1800time window here  
  dplyr::group_by(Subject, Item, condition,TRIAL_INDEX, percentile, aoa) %>%  #these define the variables you want to include in your model / average over
 #Below summarizes the data 
   dplyr::summarise(Target_P = mean(IA_Target_P),      
            Distractor_P = mean(IA_Distractor_P),
            LogGaze = mean(LogGaze))


FF_Agg_Long <- FF_ET %>% 
dplyr::filter (Time > 300 & Time < 4000) %>%                  #these numbers define your time window - we're using a standard 300-1800time window here  
  dplyr::group_by(Subject, Item, condition,TRIAL_INDEX, percentile, aoa) %>%  #these define the variables you want to include in your model / average over
 #Below summarizes the data 
   dplyr::summarise(Target_P = mean(IA_Target_P),      
            Distractor_P = mean(IA_Distractor_P),
            LogGaze = mean(LogGaze))

#Add accuracy
FF_Agg$Accuracy <-  (FF_Agg$Target_P / (FF_Agg$Target_P + FF_Agg$Distractor_P))
FF_Agg_Long$Accuracy <-  (FF_Agg_Long$Target_P / 
                            (FF_Agg_Long$Target_P + FF_Agg_Long$Distractor_P))
```

## View data across conditions

```
#summarized by subject
FF_Agg_BySubj <- FF_Agg %>% 
  dplyr::group_by(Subject, condition) %>%
  dplyr::summarize(LogGaze = mean(LogGaze),
            Accuracy=mean(Accuracy))

FF_Agg_Long_BySubj <- FF_Agg_Long %>% 
  dplyr::group_by(Subject, condition) %>%
  dplyr::summarize(LogGaze = mean(LogGaze),
            Accuracy=mean(Accuracy))
```

### Compare in 300-1800 ms time window

```
#are there differences by condition in looking by condition?
#standard window
ggwithinstats(data=FF_Agg_BySubj,
               x= condition,
              y=LogGaze,
             ylab="LogGaze",
              xlab="Condition",
              title = "Familiar Object Recognition by Condition \n(300-1800ms time window)", 
             grouping.var = "Subject",
             caption="Comparison of High and Low Sensory conditions") +
             ggplot2::scale_color_manual(values=c("#1170aa", "#fc7d0b"))
```

```
#data support difference by condition
ggsave(file="../Figs/FF_Distribution_Comparions.png", width=5.35)
```

### Compare in 300-4000 ms time window

```
#long window
ggwithinstats(data=FF_Agg_Long_BySubj,
               x= condition,
              y=LogGaze,
             ylab="LogGaze",
              xlab="Condition",
              title = "Familiar Object Recognition by Condition \n(300-4000ms time window)", 
             grouping.var = "Subject",
             caption="Comparison of High and Low Sensory conditions") +
             ggplot2::scale_color_manual(values=c("#1170aa", "#fc7d0b"))
```

```
#data support difference by condition
ggsave(file="../Figs/FF_Late_Distribution_Comparions.png", width=5.35)
```

## Statistical models

### modelling performance in 300- 1800 time window

```
#lmer model of the same effect - including subjects and items as random effects

#dfSummary(FF_Agg) in the 300-1800 time window
LogGazeFF <- lmer(LogGaze ~ condition*(scale(percentile)) + scale(aoa)  + (1|Subject) + (1|Item), FF_Agg)

#plot regression coefficient effects
plot_model(LogGazeFF)
```

```
#show regression table cleanly 
tab_model(LogGazeFF)
```

|  | LogGaze | | |
| --- | --- | --- | --- |
| Predictors | Estimates | CI | p |
| (Intercept) | 0.85 | 0.39 – 1.30 | **<0.001** |
| condition [High] | 0.67 | 0.05 – 1.29 | **0.035** |
| percentile | 0.22 | -0.05 – 0.49 | 0.112 |
| aoa | -0.08 | -0.38 – 0.23 | 0.629 |
| condition [High] \* percentile | -0.20 | -0.54 – 0.14 | 0.247 |
| Random Effects | | | |
| σ2 | 5.06 | | |
| τ00 Subject | 0.12 | | || τ00 Item | 0.20 | | || ICC | 0.06 | | || N Subject | 31 | | || N Item | 12 | | || Observations | 672 | | |
| Marginal R2 / Conditional R2 | 0.027 / 0.085 | | |

### Models for recognition in 300-4000 ms time window

```
#repeat analysis for 300-4000 ms window:  

LogGazeFF_long <- lmer(LogGaze ~ condition*(scale(percentile)) + scale(aoa) + (1|Subject) + (1|Item), FF_Agg_Long)

#pllot regression coefficient effects
plot_model(LogGazeFF_long)
```

```
#show regression table cleanly 

tab_model(LogGazeFF_long)
```

|  | LogGaze | | |
| --- | --- | --- | --- |
| Predictors | Estimates | CI | p |
| (Intercept) | 0.43 | -0.03 – 0.89 | 0.068 |
| condition [High] | 0.65 | 0.02 – 1.27 | **0.042** |
| percentile | 0.10 | -0.13 – 0.33 | 0.395 |
| aoa | -0.01 | -0.32 – 0.29 | 0.933 |
| condition [High] \* percentile | -0.15 | -0.41 – 0.11 | 0.260 |
| Random Effects | | | |
| σ2 | 2.87 | | |
| τ00 Subject | 0.17 | | || τ00 Item | 0.24 | | || ICC | 0.12 | | || N Subject | 31 | | || N Item | 12 | | || Observations | 672 | | |
| Marginal R2 / Conditional R2 | 0.033 / 0.154 | | |

There is a clear difference in looking between High and Low sensory
conditions. With looks facilitated in high sensory conditions vs. lower
sensory conditions. Lmer model also supports this conclusion across both
time windows

# Novel word analysis ( Experiment 3)

## Experiment 3: Timecourse plots

visualize the timecourse

```
NovelPlt<-ggplot(Novel_ET, aes(x=Time, y=LogGaze, group=condition, color=condition, fill=condition)) +
  labs(title = "Recognition of words across condition", x="Time in ms from noun onset") +
  geom_hline(yintercept = 0)+
  stat_summary(fun.data=mean_se, geom="ribbon", alpha=.1,color=NA,show.legend = FALSE)+
  stat_summary(fun=mean, geom="line", size=2) +
  scale_color_tableau()+
  scale_fill_tableau()+
  theme_few()+
  theme(legend.text=element_text(size=18),
        legend.title=element_blank(),
        legend.position="bottom") +
  guides(linetype=guide_legend(keywidth = 5, keyheight= 1),
         color=guide_legend(keywidth= 5, keyheight = 1))
NovelPlt
```

```
ggsave("../Figs/Novel_TC.png")
```

```
## Saving 7 x 5 in image
```

## Aggregate Novel word into time bins

Use a short time bin that is typically used in familiar word studies
(300-1800), and a longer time bin spanning the length of the trial more
commonly used in novel word studies (since the time course of novel word
recognition tends to be much slower)

```
Novel_Agg <- Novel_ET %>% 
  dplyr::filter (Time > 300 & Time < 1800) %>%                  #these numbers define your time window - we're using a standard 300-1800time window here  
    dplyr::group_by(Subject, Item, condition,TRIAL_INDEX,  percentile) %>%  #these define the variables you want to include in your model / average over
 #Below summarizes the data 
   dplyr::summarise(Target_P = mean(IA_Target_P),      
            Distractor_P = mean(IA_Distractor_P),
            LogGaze = mean(LogGaze))


Novel_Agg_Long <- Novel_ET %>% 
  dplyr::filter (Time > 300 & Time < 4000) %>%                  #these numbers define your time window - we're using a longer timewindow here, consistent with prior novel word learning windows  
    dplyr::group_by(Subject, Item, condition,TRIAL_INDEX, percentile) %>%  #these define the variables you want to include in your model / average over
 #Below summarizes the data 
   dplyr::summarise(Target_P = mean(IA_Target_P),      
            Distractor_P = mean(IA_Distractor_P),
            LogGaze = mean(LogGaze))

#Add accuracy
Novel_Agg$Accuracy <-  (Novel_Agg$Target_P / (Novel_Agg$Target_P + Novel_Agg$Distractor_P))
Novel_Agg_Long$Accuracy <-  (Novel_Agg_Long$Target_P / (Novel_Agg_Long$Target_P + Novel_Agg_Long$Distractor_P))
```

## View data across conditions

### Compare first in 300-1800 ms window

Recently mapped novel words typically are recognized more slowly than
the typical “familiar” word timecourse. Therefore this time window is
not reported in the ms and only included for completeness.

```
## View shape of data

#summarized by subject
Novel_Agg_BySubj <- Novel_Agg %>% 
  dplyr::group_by(Subject, condition) %>%
  dplyr::summarize(LogGaze = mean(LogGaze),
            Accuracy=mean(Accuracy))

Novel_Agg_Long_BySubj <- Novel_Agg_Long %>% 
  dplyr::group_by(Subject, condition) %>%
  dplyr::summarize(LogGaze = mean(LogGaze),
            Accuracy=mean(Accuracy))

#are there differences by condition in looking by condition?
#standard window
ggwithinstats(data=Novel_Agg_BySubj,
               x= condition,
              y=LogGaze,
             ylab="LogGaze",
              xlab="Condition",
              title = "Novel Object Recognition by Condition (300-1800ms)", 
             grouping.var = "Subject",
             caption="Comparison of High and Low Sensory conditions") +
             ggplot2::scale_color_manual(values=c("#1170aa", "#fc7d0b"))
```

```
#early differences across condition not visually apparent - but not expected for novel word recognition
ggsave(file="../Figs/Novel_Early_Distribution_Comparions.png", width=5.35)
```

### Compare novel word recognition in 300-4000 ms time window

```
#longer time window 
ggwithinstats(data=Novel_Agg_Long_BySubj,
               x= condition,
              y=LogGaze,
             ylab="LogGaze",
              xlab="Condition",
              title = "Novel Object Recognition by Condition (300-4000 ms)", 
             grouping.var = "Subject",
             caption="Comparison of High and Low Sensory conditions") +
             ggplot2::scale_color_manual(values=c("#1170aa", "#fc7d0b"))
```

```
#visual patterns illustrate a different, but not significant in aggregate 
ggsave(file="../Figs/Novel_Late_Distribution_Comparions.png", width=5.35)
```

## Statistical models

### Comparing in 300-4000 ms window

```
#lmer model of the same effect - including subjects and items as random effects,  and accounting for variance related to vocabulary size

LogGazeNovel <- lmer(LogGaze ~ condition*(scale(percentile)) + (1|Subject) + (1|Item), Novel_Agg_Long)


#table of regression modely cleanly
tab_model(LogGazeNovel)
```

|  | LogGaze | | |
| --- | --- | --- | --- |
| Predictors | Estimates | CI | p |
| (Intercept) | -0.19 | -0.86 – 0.47 | 0.568 |
| condition [Touch] | 0.43 | 0.05 – 0.81 | **0.028** |
| percentile | 0.06 | -0.23 – 0.36 | 0.684 |
| condition [Touch] \* percentile | 0.19 | -0.20 – 0.58 | 0.334 |
| Random Effects | | | |
| σ2 | 2.09 | | |
| τ00 Subject | 0.09 | | || τ00 Item | 0.18 | | || ICC | 0.12 | | || N Subject | 30 | | || N Item | 2 | | || Observations | 221 | | |
| Marginal R2 / Conditional R2 | 0.033 / 0.144 | | |

```
#plot regression coefficient effects
plot_model(LogGazeNovel)
```

```
#this model indicates that the object initially exposed in the high sensory condition (touch) was better recognized than that in the lower sensory condition.    when accounting for random variance of subjects, items,  and fixed effects of vocabulary percentile
```

## SAVE

```
save.image('../Output/Exp2&3_Analysis.Rdata')
```
